# Rombocin, a Short Stable Natural Nisin Variant, Displays Selective Antimicrobial Activity against *Listeria monocytogenes* and Employs a Dual Mode of Action to Kill Target Bacterial Strains

**DOI:** 10.1021/acssynbio.3c00612

**Published:** 2024-01-09

**Authors:** Longcheng Guo, Joseph Wambui, Chenhui Wang, Jaap Broos, Roger Stephan, Oscar P. Kuipers

**Affiliations:** † Department of Molecular Genetics, Groningen Biomolecular Sciences and Biotechnology Institute, University of Groningen, Groningen 9747 AG, The Netherlands; ‡ Institute for Food Safety and Hygiene, Vetsuisse Faculty, University of Zurich, Zurich 8057, Switzerland

**Keywords:** short nisin variant, four lanthionine rings, mode of action, stability, specificity, mutagenesis

## Abstract

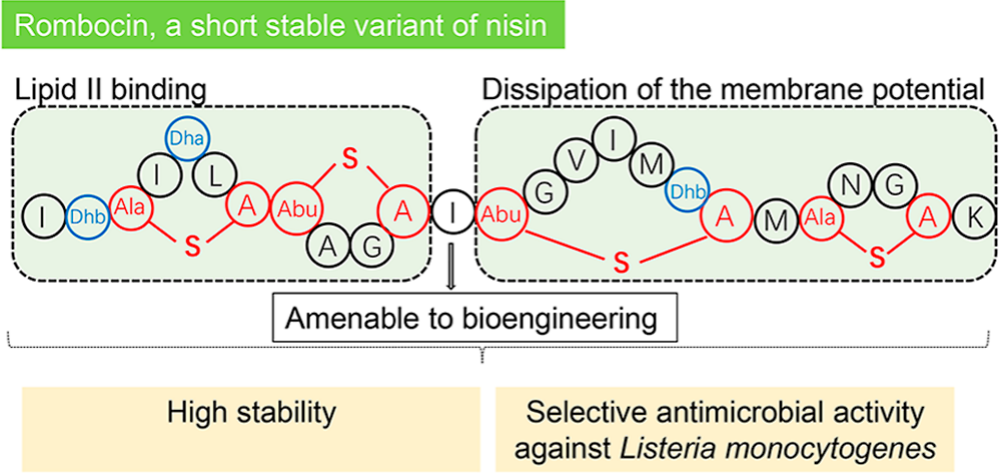

Nisin, with its unique mode of action and potent antimicrobial activity, serves as a remarkable inspiration for the design of novel antibiotics. However, peptides possess inherent weaknesses, particularly their susceptibility to proteolytic degradation, such as by trypsin, which limits their broader applications. This led us to speculate that natural variants of nisin produced by underexplored bacterial species can potentially overcome these limitations. We carried out genome mining of two *Romboutsia sedimentorum* strains, RC001 and RC002, leading to the discovery of rombocin A, which is a 25 amino acid residue short nisin variant that is predicted to have only four macrocycles compared to the known 31–35 amino acids long nisin variants with five macrocycles. Using the nisin-controlled expression system, we heterologously expressed fully modified and functional rombocin A in *Lactococcus lactis* and demonstrated its selective antimicrobial activity against *Listeria monocytogenes*. Rombocin A uses a dual mode of action involving lipid II binding activity and dissipation of the membrane potential to kill target bacteria. Stability tests confirmed its high stability at different pH values, temperatures, and in particular, against enzymatic degradation. With its gene-encoded characteristic, rombocin A is amenable to bioengineering to generate novel derivatives. Further mutation studies led to the identification of rombocin K, a mutant with enhanced bioactivity against *L. monocytogenes*. Our findings suggest that rombocin A and its bioengineered variant, rombocin K, are promising candidates for development as food preservatives or antibiotics against *L. monocytogenes*.

## 1 Introduction

The escalating rates of morbidity and mortality attributed to antibiotic-resistant bacteria pose an increasing threat to global health. Projections in recent studies indicate that, without targeted intervention strategies, annual global deaths resulting from drug-resistant infections could surge to 10 million by the year 2050.^1^ In this context, there is an unmistakable and urgent need for novel classes of antibiotics that can function through previously unexplored mechanisms. Bacteriocins, small antimicrobial peptides produced by various bacterial species, represent a compelling resource in response to these challenges.^2^ Nisin, a 34-amino acid Class I lantibiotic primarily produced by *Lactococcus lactis* subspecies lactis, stands as the most extensively utilized bacteriocin.^3−5^ Nisin holds immense promise for therapeutic applications, effectively combatting various Gram-positive antibiotic-resistant organisms such as vancomycin-resistant *Enterococcus* and methicillin-resistant *Staphylococcus aureus*.^5^ Its rarity in developing resistance can be attributed to its dual inhibition mechanisms: binding to lipid II, disrupting cell wall biosynthesis, and forming membrane pores, resulting in cellular component leakage.^6^ However, the peptidic composition of nisin entails certain limitations. Of particular limitation is its susceptibility to proteolytic degradation in vivo.^7^ Several approaches have been developed to enhance the stability and efficacy of nisin, such as encapsulation, and combination with other antimicrobials.^8^ However, these methods do not fundamentally address nisin’s sensitivity to proteolytic degradation. It has been shown that nisin most degradation occurring in the C-terminal region.^9,10^ Previous findings demonstrated that degradation fragments (e.g., nisin1–29, and nisin1–32) exhibited low antibacterial activity.^9^ Therefore, the identification of short natural nisin variants that lack part of or even the full C-terminal region while retaining antimicrobial potency offers an attractive alternative to overcome these current limitations of nisin. These new variants can also be developed through bioengineering to further enhance their properties.

**Figure 1:**
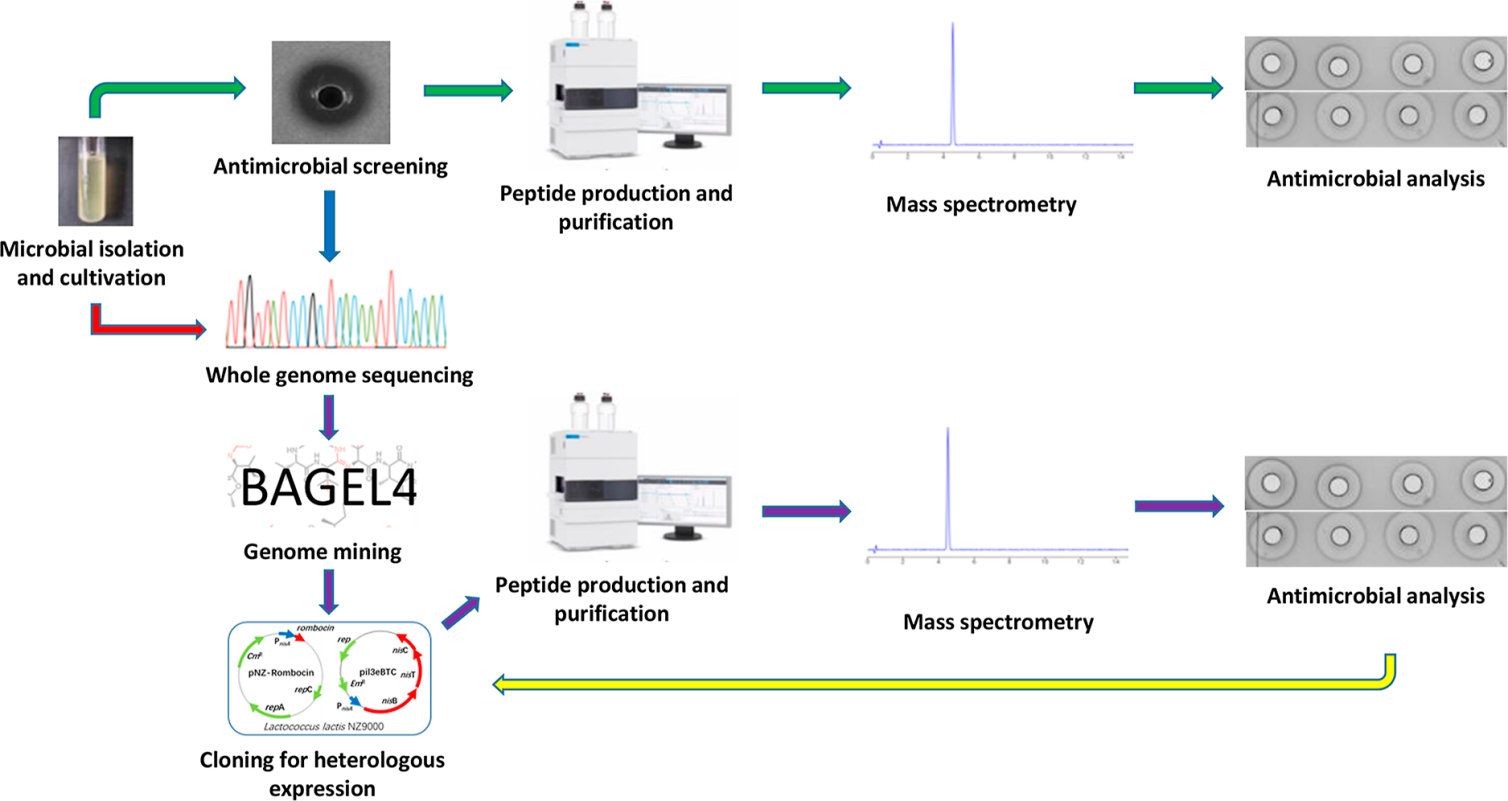
Scheme of two major approaches involved new lantibiotic discovery and characterization. In the first approach (shown in green arrow), bacteriocin producing strains are identified through culture-based approaches. The second approach involves first the whole genome sequencing of bacterial isolates. This is followed by detection of bacteriocin biosynthetic gene clusters through genome mining tools such as BAGEL4. Identified bacteriocins are cloned into suitable expression systems such as nisin-controlled gene expression (NICE) system for expression in suitable host such as *L. lactis*. The culture-based and genome-based approaches then incorporate similar downstream processes involving peptide purification, mass and structural analysis, and antimicrobial activity analysis. A major advantage of the second approach is that it allows identified bacteriocins to be bioengineered for improved antimicrobial and physicochemical properties as shown by the yellow arrow. To avoid rediscovery of already known bacteriocins and take advantage of bioengineering strategies of the second approach, culture-based approaches can be linked to the later approach as shown by the blue arrow.

Conventionally, culture-based techniques have been used to discover natural variants of nisin and other new bacteriocins (Figure 1).^11^ However, these techniques have limitations, which include laborious processes, low bacteriocin production under standard laboratory culturing conditions, and an increasing probability of rediscovering already known bacteriocins. To overcome these challenges, genome-guided methods that detect biosynthetic gene clusters (BGCs) that may encode novel nisin variants can be applied due to their numerous advantages. First, they allow research efforts to be concentrated on unknown BGCs, especially those derived from underexplored microbes. Second, the majority of the underexplored bacteria are either unculturable or difficult to cultivate under standard laboratory conditions, with the latter group often requiring external stimuli to express their BGCs. Identification of essential biosynthetic genes through genome mining-based approaches offers a basis for development of heterologous approaches that facilitate expression and subsequent characterization of compounds encoded in these novel BGCs (Figure 1).^12^ The genome-guided approach can also facilitate the design of novel peptides using the discovered bacteriocins as a template for improved antimicrobial or physicochemical properties (Figure 1).

Nisin-like BGCs encode at least one peptide precursor.^13^ Other genes include a dehydratase (LanB) that is involved in the dehydration of serines and threonines and a cyclase (LanC) that is involved in cyclization of the dehydrated amino acids to Cys, creating either lanthionine or methyllanthionine. Also encoded is a dedicated ABC transporter (LanT) that recognizes a conserved signal sequence to export the prelantibiotic out of the cell and a signal protease (LanP) that cleaves off the leader peptide to activate the lantibiotic. Also present are genes encoding self-immunity proteins (LanFEG and in other cases LanI) and regulatory proteins (LanRK) to regulate lantibiotic biosynthesis, respectively.^14^ The fact that not all encoded genes are essential for the biosynthesis of lantibiotics means that BGCs can easily be refactored to only include essential biosynthetic genes, thus overcoming the laborious expression of the gene cluster and/or modification enzymes. Furthermore, it has been shown that a *L. lactis* strain, coexpressing a peptide with nisBTC can produce and export not only fully modified peptide but also a non-lanthipeptide fusion of the leader peptide with dehydrated angiotensin.^15^ The broad substrate specificity of the nisin dehydrating and transport machinery (NisBTC) indicates the potential utilization of lantibiotic enzymes for synthesizing a diverse array of peptides containing novel dehydrated residues or novel lantibiotic structures.

**Figure 2:**
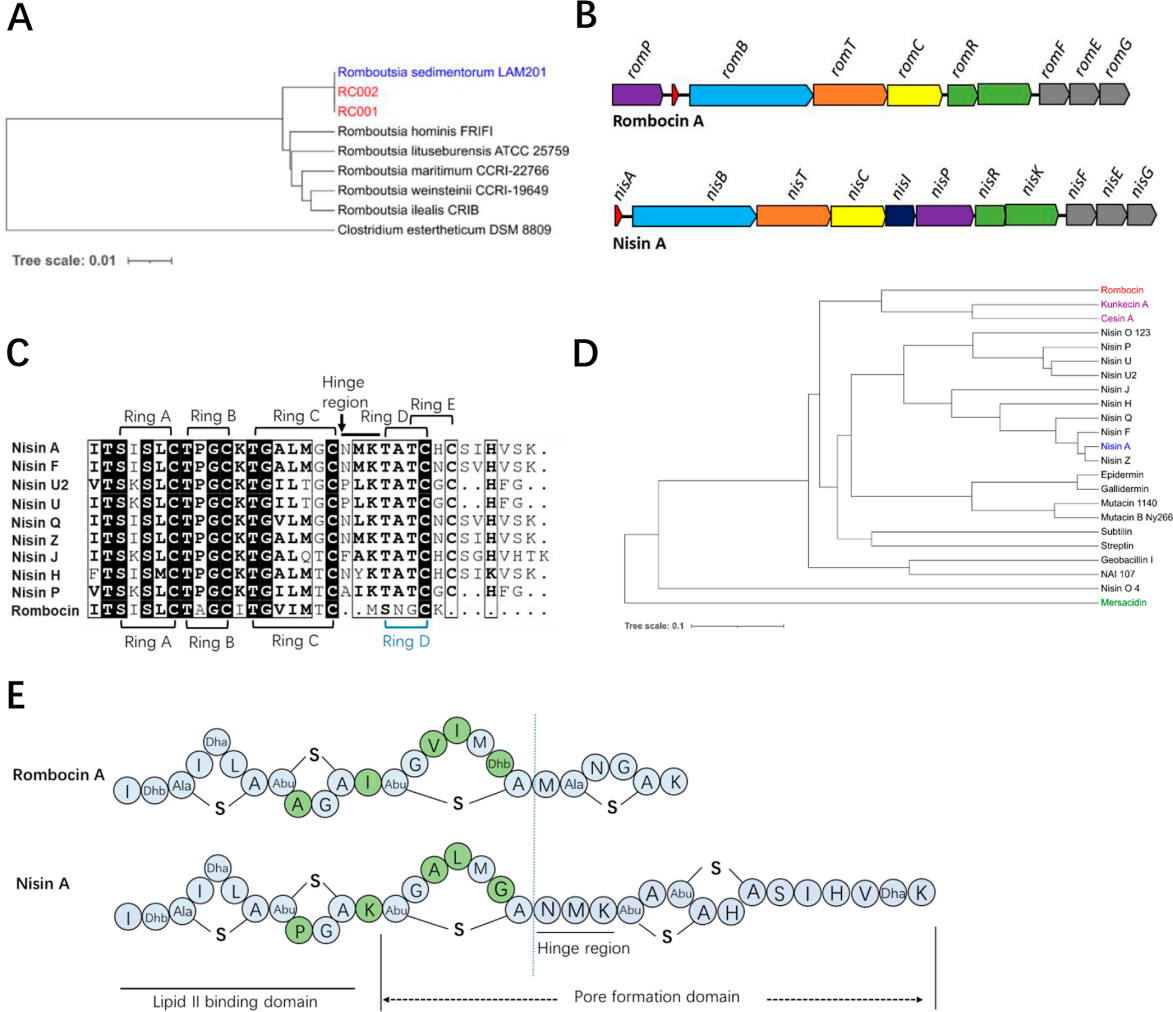
Rombocin A is the first bacteriocin discovered in the *Romboutsia* genus. (A) Two strains, RC001 and RC002, isolated from chilled vacuum packed meat were identified as members of species *Romboutsia sedimentorum* as shown by a phylogenetic tree created from the alignment of 16S rRNA sequences (1216–1217 bp) of the two strains and types strains of known species within the *Romboutsia* genus. (B) Genome mining identified a class I lanthipeptide gene cluster in the genome of *R. sedimentorum* RC002. The encoded bacteriocin has been named rombocin A. The gene cluster of rombocin A encodes the precursor peptide, *romA*, biosynthetic genes, *romBTC*, regulatory genes, *romRK*, immunity genes, *romFEG* and maturation protease, *romP*. Genes with similar functions are present in the biosynthetic gene cluster of nisin A as shown through color coding. (C) Amino acid sequences alignment of rombocin A and nisin variants. Gray, similar residues; black, identical residues. The rings and hinge region (NMK) are indicated. (D) Phylogenetic analysis of rombocin A and nisin variants. The order in which they branch shows the relatedness between them, and the branch length represents the phylogenetic distance (0.05 represents a scale for the phylogenetic distance). (E) The alignment of the amino acid sequences of rombocin A and nisin A. The functional domains, including lipid II binding site, pore formation domain, and hinge region are indicated. The first three rings of rombocin A differ from nisin A, as indicated by green-highlighted amino acid differences.

Recently, anaerobic bacteria have attracted attention as novel sources of antimicrobial compounds.^16^ Genome-wide studies within the *Clostridium* genus led to the identification of BGCs for Class I lanthipeptides^17^ leading us to hypothesize that some anaerobic bacteria have evolved to produce natural nisin variants or other Class I lanthipeptides that display changed and sometimes improved properties, such as improved solubility, stability, or specificity. In our efforts to identify novel natural nisin variants or nisin-like bacteriocins in anaerobic bacteria, we made the discovery of rombocin A, an unusual short natural nisin variant, encoded in the genome of the psychotropic and anaerobic growing *Romboutisia sedimentorium* RC002 through genome mining (Figure 2). In this study, we present the in vitro production of rombocin A using a modified nisin-controlled gene expression (NICE) system. The fully modified peptide was synthesized, and its antimicrobial properties, stability under different conditions, and mode of action were investigated to assess its potential as a biopreservative or drug agent. Additionally, we performed mutation studies and identified a mutant with a further enhanced bioactivity.

## 2 Results

### 2.1 Genome Mining-Based Identification of Rombocin A in the Genome of *R. sedimentorum* RC002

#### 2.1.1 Strains RC001 and RC002 Belong to Species *R. sedimentorum*

The draft genome sequences of two new isolates, RC001 (Ref_seq GCA_030131965.1) and RC002 (Ref_seq GCA_030131845.1), isolated from chilled vacuum-packed meat were assembled into 36 and 71 contigs, and their sizes were 3.5 and 3.3 Mbp, respectively. The guanine-cytosine (GC) content of both genomes was 28.0%. In silico-based identification of both strains using the 16S rRNA sequences extracted in silico from respective genomes revealed they were 99.9% identical to *R. sedimentorum* LAM201^T^, the type strain of recently identified species within the *Romboutsia* genus.^18^ Further 16S rRNA sequence-based phylogenetic analysis using other members of the *Romboutsia* genus confirmed both RC001 and RC002 were members of species *R. sedimentorum* (Figure 2A). Consequently, the strains were named *R. sedimentorum* RC001 and *R. sedimentorum* RC002, respectively.

#### 2.1.2 Genome of Strain RC002 Encodes a Short Nisin Variant, Rombocin A

Genome mining using antiSMASH and BAGEL4 identified a 12.9 kb Class I lanthipeptide gene cluster in the genome of *R. sedimentorum* RC002. The gene cluster consists of 10 genes compared to 11 genes in the nisin A gene cluster^14^ (Figure 2B). Consistent with the nomenclature for Class I lanthipeptides, these genes are *romPABTCRKFEG* with *romA* being the structural gene. *RomA* was predicted to encode a 25 amino acid core peptide that is shorter than the 31–35 amino acids of other natural nisin variants (Figure 2C). A phylogenetic tree of known Class I lanthipeptides confirmed that rombocin A belonged to the same class (Figure 2D). Rombocin A is therefore a novel member of the ever growing family of nisin-like bacteriocins. Due to its short sequence and lack of the fifth macrocycle that is characteristic of many published nisin variants (Figure 2C) as well as its phylogenetic relatedness to other Class I lanthipeptides, kunkecin A and cesin A, the encoded bacteriocin is here referred to as rombocin A, referring to a “*Romboutsia* spp. bacteriocin”. Compared to nisin A, rombocin A differs by five amino acids, namely, Pro9Ala, Lys12Ile, Ala15Val, Leu16Ile, and Gly18Thr, all located in rings B and C (Figure 2E).

### 2.2 Rombocin A is Fully Modified Using the Promiscuous Nisin Modification Machinery

Nisin’s antibacterial activity is attributed to the insertion of its two C-terminal macrocycles, known as rings D and E, into the cell membrane, leading to pore formation in target bacteria. This insertion process involves the reorientation of nisin, which is facilitated by the hinge region (NMK) located between rings C and D (Figure 2E).^19^ Interestingly, despite the critical role of these structural features in nisin’s antibacterial activity, rombocin A lacks both the hinge region and one of the five macrocycles forming ring E. These features led us to theorize that rombocin A could have quite different properties than nisin, which necessitated further investigation.

**Figure 3:**
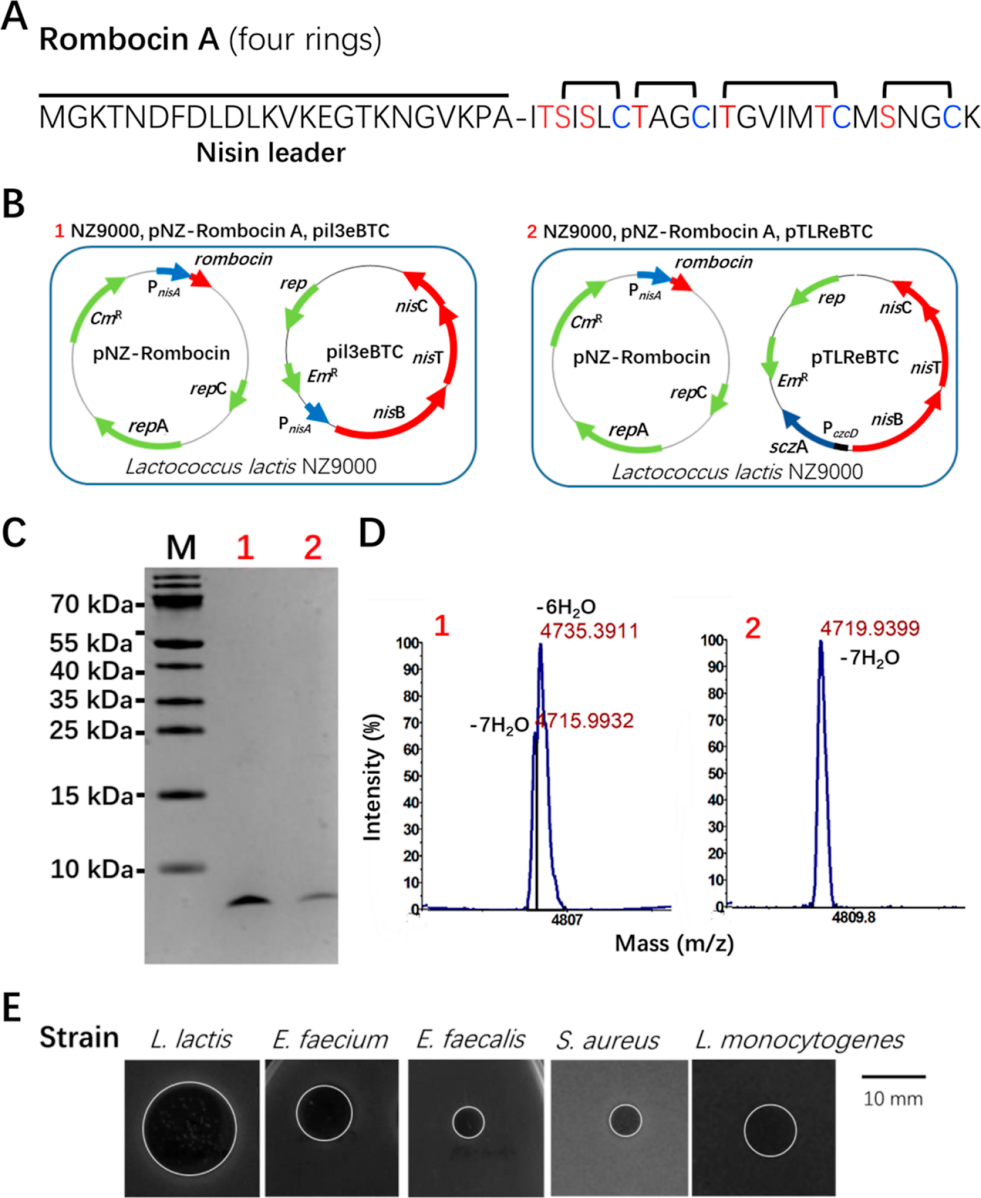
Heterologous expression of rombocin in *L. lactis* using the nisin modification machinery NisBTC. (A) The heterologous expression of rombocin core peptide using nisin leader peptide. (B) The new developed nisin-controlled gene expression (NICE) system. The traditional system involves two plasmids, pil3eBTC and pNZ-rombocin, where the modification machinery *nisBTC* and *rombocin A* are induced simultaneously by nisin. In contrast, our novel system utilizes a two-step induction process, where *nisBTC* is first induced by Zn^2+^ and nisin is added 3 h later. The *rombocin*, encoding the peptide rombocin with nisin leader attached; *sczA*, encoding the repressor of P_*czcD*_; P_*czcD*_, a Zn^2+^ inducible promoter; P_*nisA*_, a nisin inducible promoter; *nisB*, *nisT*, and *nisC*, encoding nisin modification machinery NisBTC; *rep*, encoding plasmid replication protein; *Cm*^R^, chloramphenicol resistance gene; and *Em*^R^, erythromycin resistance gene. (C) Tricine-SDS-PAGE analysis of peptide expression using the two expression systems. Each lane contained peptides from 0.2 mL of supernatant. (D) MALDI-TOF MS analysis to evaluate the dehydration efficiency of the peptides expressed using both systems. (E) Screening the antibacterial activity of peptides after cleavage of nisin leader part using NisP. The white circle indicates the antibacterial halo caused by the rombocin peptide, leading to the inhibition of bacterial strain growth.

We first tried using the well-studied and promiscuous nisin A synthetic machinery^20^ for the heterologous expression of rombocin A. The core peptide of rombocin A was fused to the nisin A leader peptide (Figure 3A), where the leader’s function is to guide the core peptides through intricate postmodification processes catalyzed by the modification machinery NisBC, and export outside the cell by NisT (Figure 3B). Tricine-SDS page analysis confirmed expression of the expected rombocin A fusion peptide (Figure 3C), and MALDI-TOF MS analysis detected two major peaks at 4715 and 4735 Da, corresponding to 7 times and 6 times dehydrated rombocin A, respectively (Figure 3D1 and Table S1). This demonstrated that NisB can fully dehydrate the bacteriocin. However, purification of rombocin A from 1 L medium was difficult due to the presence of a mixture of peptides that had different levels of dehydration (Figure S1). In the traditional nisin expression system, both *nisA* and *nisBTC* genes are controlled by the P_*nisA*_ promoter in separate plasmids (Figure 3B1). To improve the modification efficiency, we hypothesized that inducing the NisBTC modification enzymes before peptide induction might enhance the rombocin A modification (Figure 3B2). A two-step induction process was employed, where nisBTC is first induced by Zn^2+^ and nisin is added 3 h later to start the expression of the prepeptide rombocin. Indeed, when we induced *nisBTC* prior to the peptide production, we still detected the expression of the lanthipeptide in the Tricine-SDS page gel (Figure 3C). Furthermore, MALDI-TOF MS analysis detected a major peak at 4719 Da (Figure 3D2 and Table S1), which is the expected mass of fully dehydrated rombocin A.

The antimicrobial activity of expressed rombocin A was evaluated against five different strains by measuring the inhibition zones on agar plates supplemented with NisP (Figure 3E), confirming that NisP effectively cleaved the nisin leader peptide. The core peptide was consequently removed using NisP and purified by HPLC (Figure S2). Rombocin A consists of four macrocycles, as shown in Figure 2E. To confirm the macrocyclization of the lanthipeptide, we performed an *N*-ethylmaleimide (NEM) alkylation assay. The major peak detected in the MALDI-TOF MS analysis had a mass of 2380 Da, corresponding to fully dehydrated rombocin A, and no mass shift of 125 Da was observed following NEM incorporation in any of the four cysteines of rombocin (Figure S2A,B). These results demonstrate that rombocin A was fully cyclized by NisC. Based on the highly similar amino acid sequence to nisin and its variants, along with fully dehydrated Ser/Thr and the absence of free Cys, we illustrate the proposed structure of rombocin in Figure 2E.

### 2.3 Mutant I/K (Rombocin K) Improves the Antibacterial Activity

**Figure 4:**
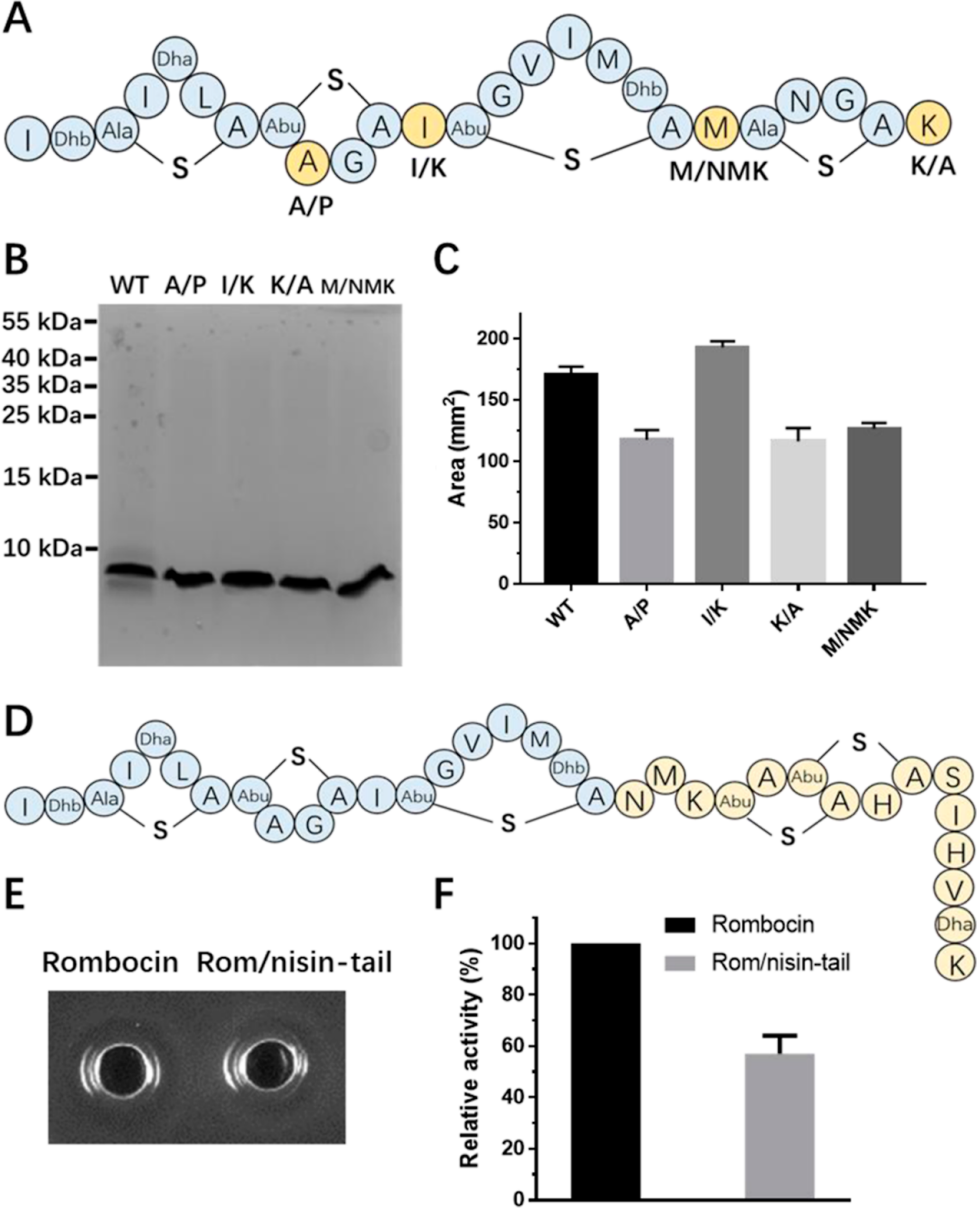
Antimicrobial activity of rombocin and its analogues against *L. monocytogenes* LMG10470. (A) The structures of wild-type (WT) rombocin and four bioengineered analogues, namely, rombocin A/P, rombocin I/K (referred to as rombocin K), rombocin M/NMK, and rombocin K/A, which were generated through amino acid substitutions at positions A9, I12, M20, and K25, respectively, as indicated by the yellow residues. (B) Tricine-SDS-PAGE gel analysis of the purified rombocin and its analogues. (C) Relative antimicrobial activity of the four rombocin analogues and wild-type rombocin against *L. monocytogenes*. (D) The structure of the bioengineered rombocin analog fused with the nisin A pore-forming domain (denoted as rom/nisin-tail). (E) Antimicrobial activity of the wild-type rombocin and rom/nisin-tail against *L. monocytogenes*. (F) Relative antimicrobial activity of the wild-type rombocin and rom/nisin-tail against *L. monocytogenes*.

Comparing the amino acid sequences of nisin variants and rombocin A (Figure 2C), we found two residues that are conserved in all nisin variants but absent in rombocin: A9 and I12, which correspond to P9 and K12 in nisin. In addition, the hinge region (NMK) of nisin, which has a profound influence on antimicrobial activity and host specificity,^21^ contains only one amino acid (Met) in rombocin A. We also hypothesized that the terminal Lys plays a functional role, as it is present in most lantibiotics. To determine the functional roles of these three amino acids and hinge region, we created analogs of rombocin A with substitutions at these positions (Figure 4A). Among these analogs, I/K (referred to as rombocin K) shows a significant increase (13%) in antimicrobial activity against *L. monocytogenes*, while A/P, K/A and M/NMK all showed reduced activity (25–31%) (Figure 4B,C). On the other hand, when we attached the nisin rings D and E to rombocin (Figure 4D), the full nisin mimic exhibited a dramatic decrease in activity (−40%) (Figure 4E,F).

### 2.4 Rombocin Displays Selective Antimicrobial Activity against *L. monocytogenes*

The antibacterial activity of rombocin A and its variant rombocin K was evaluated by using a minimal inhibitory concentration (MIC) assay against 10 Gram-positive bacterial strains. Although rombocin lacks ring E and a portion of the C-terminal tail compared to nisin (Figure 2E), both peptides displayed potent activity against Gram-positive pathogens, including multidrug-resistant strains (Table 1). However, nisin was found to be more active against most target bacteria than rombocin A, with the MIC value of nisin being 40 times lower than that of rombocin against *L. lactis*. Notably, nisin also exhibited higher antimicrobial activity against *Enterococcus faecium* (32 times), as well as a 16-fold lower MIC value against *Enterococcus faecalis* and *S. aureus*. These results are consistent with nisin’s broad antimicrobial activity. Surprisingly, nisin only showed a 4-fold lower MIC value against *Bacillus cereus* and a 2-fold lower MIC value against one *Listeria* strain (*L. monocytogenes* LK132). The efficacy of rombocin A can be improved through bioengineering, as evidenced by rombocin K. This mutant exhibited increased activity compared to the wild-type rombocin against all tested *Listeria* strains, with a 2-fold lower MIC value. Notably, rombocin K even demonstrated effectiveness comparable to that of nisin against one of the *Listeria* strains (*L. monocytogenes* LK132). The results of both natural and bioengineered variants of rombocin demonstrate its selective antimicrobial activity against pathogenic *L. monocytogenes*.

**Table 1:** Antimicrobial Profile of Rombocin against Selected Gram-Positive Strains in Comparison to Nisin^a^

	MIC (μg/mL)		
organism and type	nisin Z	rombocin A	rombocin K
*L. lactis* MG1363	0,02	0,78	0,78
*L. monocytogenes* LMG10470	3,13	25	12,5
*L. monocytogenes* TT82E	1,56	12,5	6,25
*L. monocytogenes* LK132	12,5	25	12,5
*Bacillus cereus* CH-85	6,25	25	25
*S. aureus* LMG10147	6,25	100	100
*S. aureus* LMG15975 (MRSA)	6,25	100	100
*E. faecium* LMG11423	3,13	100	50
*E. faecium* LMG16003 (VRE)	3,13	100	50
*E. faecalis* LMG16216 (VRE)	3,13	50	50

a VRE, vancomycin-resistant *Enterococci*; MRSA, methicillin-resistant *S. aureus*.

### 2.5 Mode of Action of Rombocin

#### 2.5.1 Rombocin A Binds to Cell Wall Synthesis Precursor Lipid II and Lipoteichoic Acid

**Figure 5:**
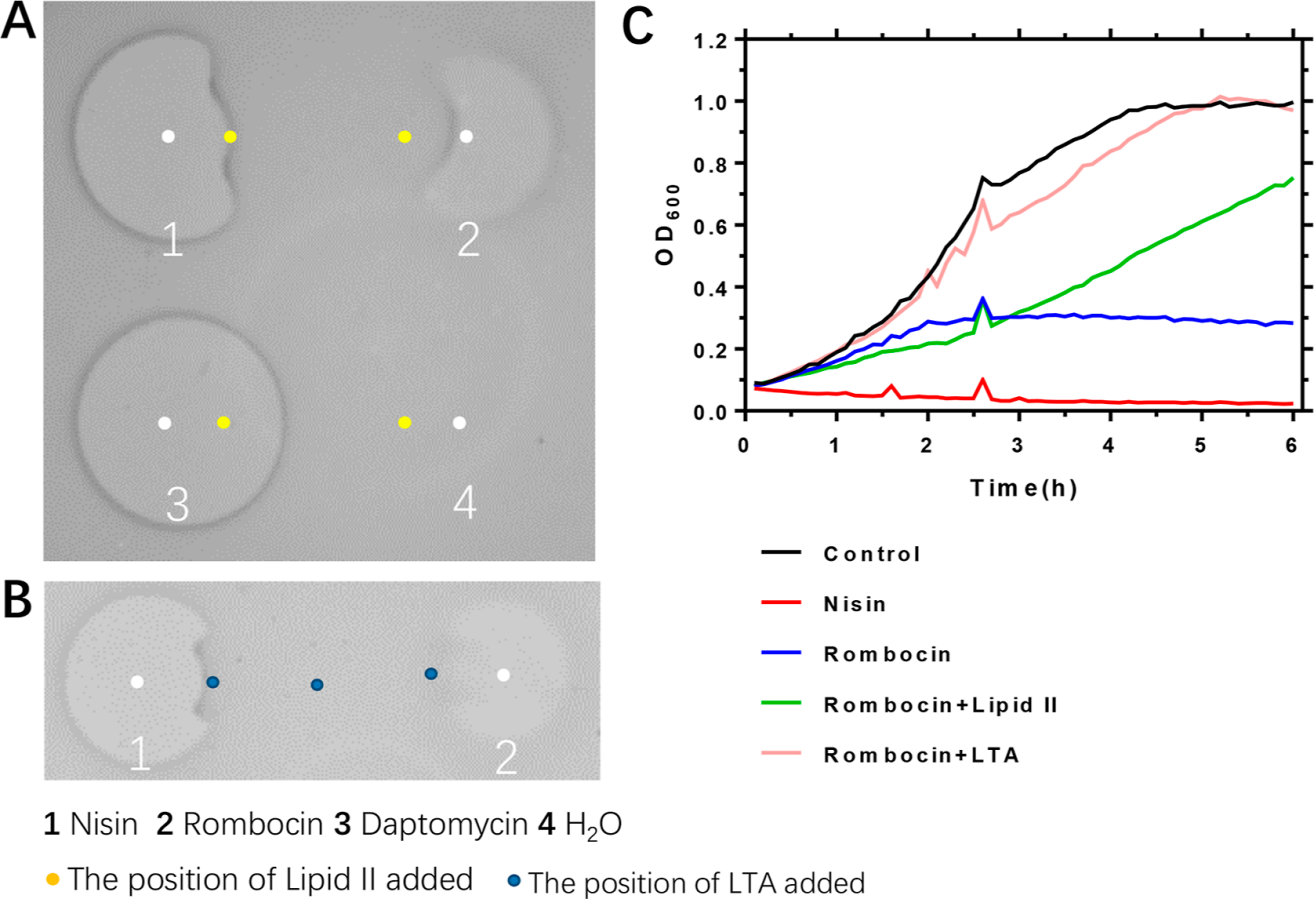
Rombocin A binds to cell wall synthesis precursor lipid II and lipoteichoic acid (LTA). (A) A spot-on-lawn assay to assess the ability of rombocin to bind to the cell wall synthesis precursor lipid II. Nisin was used as the positive control, and daptomycin and water used as the negative control. (B) A spot-on-lawn assay to investigate the binding of rombocin to the LTA. Nisin was used as the positive control. (C) Growth curve-based binding assay to determine if rombocin binds to the cell wall synthesis precursor lipid II and LTA. The small peak at 2.5 h was caused by a slight interruption when turning off the VarioskanTM LUX microplate reader.

The primary mechanism of action of lanthipeptides is the inhibition of peptidoglycan synthesis by binding to lipid II. Nisin accomplishes this through the lipid binding domain formed by rings A and B. Rombocin A shares a highly conserved lipid II binding domain with nisin, differing in only one amino acid within 11 residues (Figure 2E). To determine whether rombocin A uses a similar mode of action, we first examined its ability to bind to lipid II. This was confirmed by a reduction in the antimicrobial activity against *L. lactis* (Figure 5A). Similar observations were made for nisin, but not for daptomycin, which does not involve lipid II binding. The addition of lipid II to rombocin A also reduced its activity, as shown by the growth curves (Figure 5C). Notably, rombocin A has a net positive charge due to the presence of one positively charged amino acid at the end tail, similar to other natural nisin variants. This characteristic modulates the sensitivity of target Gram-positive bacteria through electrostatic interactions with negatively charged teichoic acids. The lipoteichoic acid (LTA) binding assay (Figure 5B) and growth curves (Figure 5C) also demonstrated that rombocin A utilizes electrostatic interactions to interact with the cell walls of target bacteria.

#### 2.5.2 Rombocin A Exerts Bactericidal Activity

**Figure 6:**
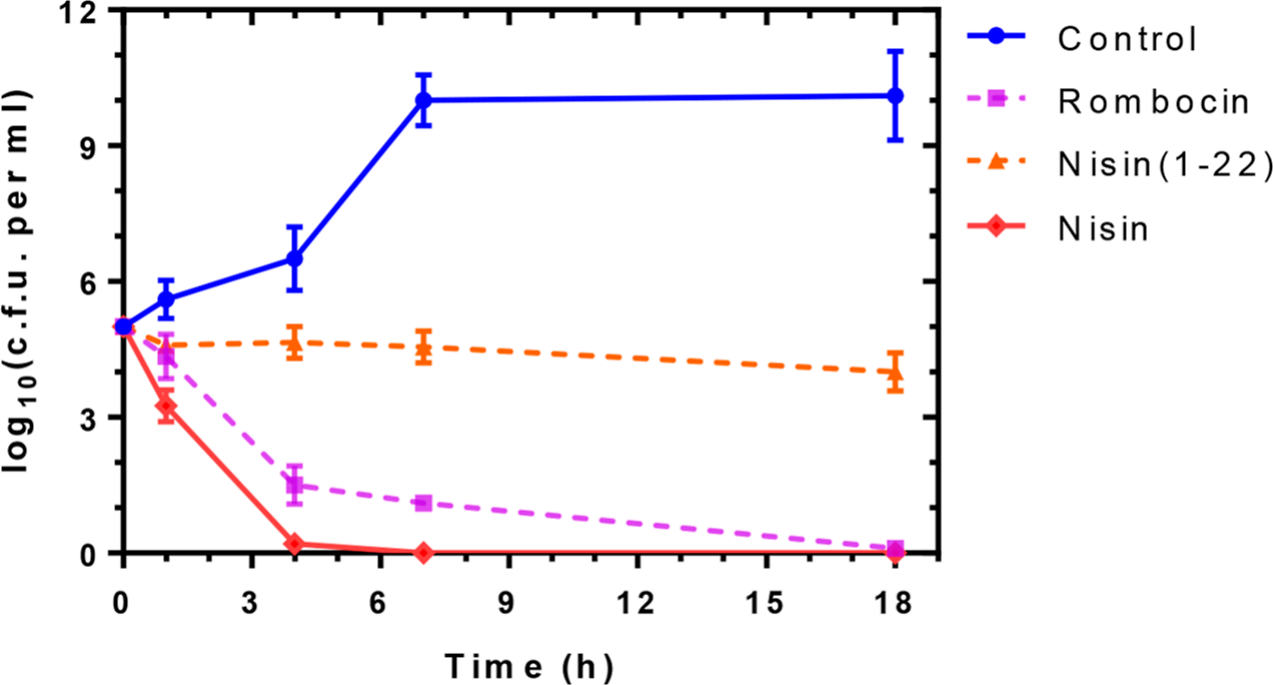
Time-dependent killing assay to determine the bacteriostatic or bactericidal activity of rombocin. 5-fold MIC of the lantibiotics was used to against *L. lactis*, along with nisin as a bactericidial control, and nisin(1–22) as a bacteriostatic control. The experiment was repeated three times, and standard deviation (SD) was calculated.

Nisin is a potent lantibiotic due to its ability to form pores in the target cell membrane and inhibit cell wall synthesis. A truncated nisin variant, nisin(1–22), has a bacteriostatic effect as it can only bind to lipid II and halt cell growth without killing the cells. To determine whether rombocin A is bacteriostatic or bactericidal, we measured its time-dependent killing kinetics and compared them to those of nisin and nisin(1–22) against *L. lactis* cells. The results showed that rombocin A caused complete killing at 18 h of incubation (Figures 6 and S3), indicating that it not only halts cell division like nisin(1–22) but also reduces the population of viable bacterial cells. Nisin acted faster than rombocin A, significantly reducing the population of viable cells until all cells were completely killed within 4 h. Our findings suggest that rombocin A has a strong bactericidal activity against bacterial cells, although cell death is not as rapid as with nisin.

#### 2.5.3 Rombocin A Impairs Membrane Functions Despite Lacking One Macrocycle in the C-Terminal Domain

**Figure 7:**
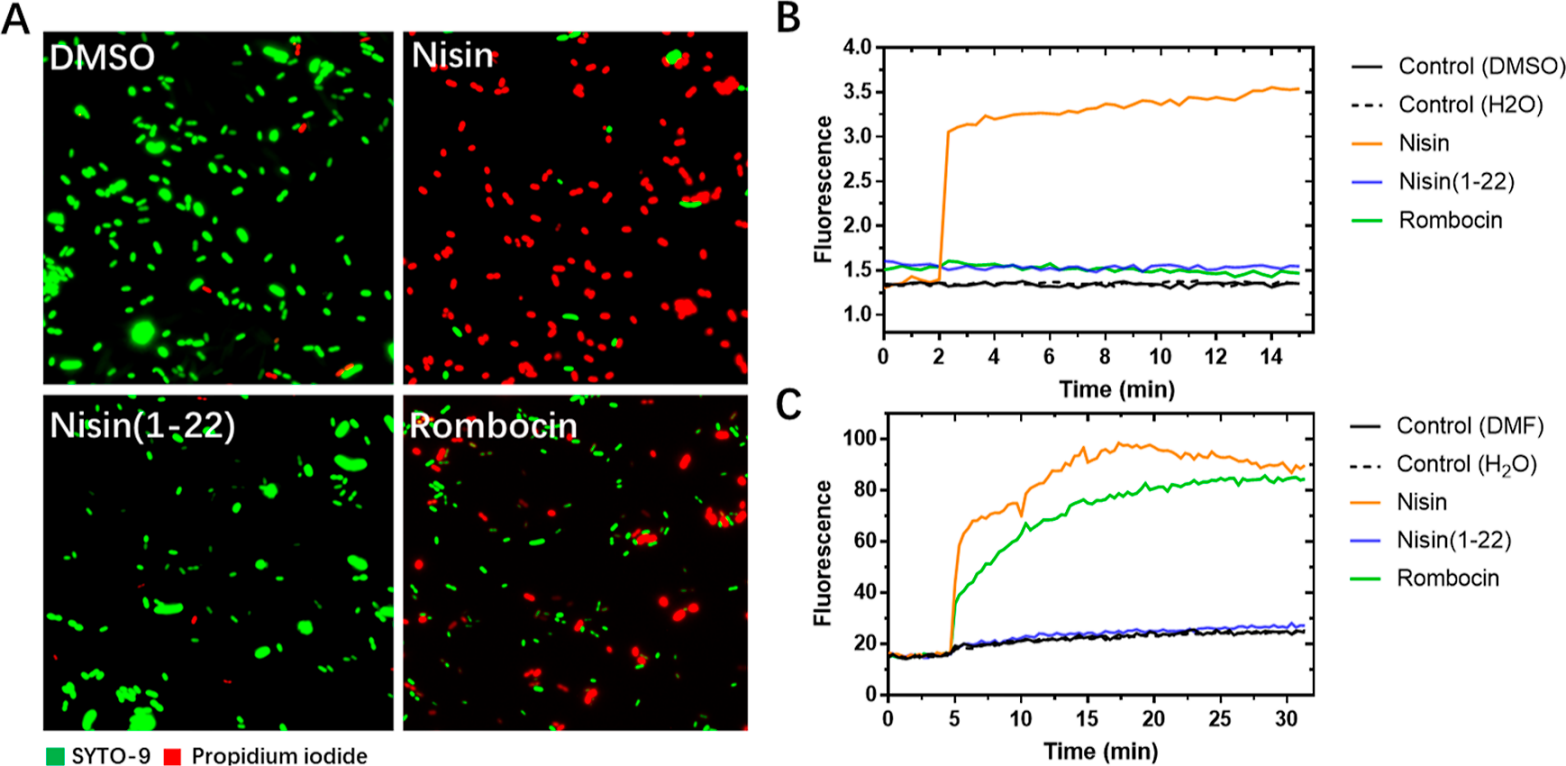
Effect of rombocin on the cellular membrane. (A) Fluorescence microscope pictures of *L. lactis* treated by 5× MIC antimicrobials for 15 min and stained with membrane-permeable SYTO-9 and membrane-impermeable propidium iodide stains. (B) Potassium leakage, as detected by the increase in fluorescence of the PBFI probe, after the addition of 5× MIC antimicrobials. At 2 min, antibiotics were added. (C) Changes in membrane potential of *L. lactis* as indicated by the increase in the fluorescence of DiSC3(5) probe after treatment of cells with 5× MIC antimicrobial. At 5 min, antimicrobial peptides were added.

To evaluate the effects of rombocin A on bacterial cell membranes, we monitored membrane integrity using green fluorescent dye SYTO 9 and red fluorescing propidium iodide (PI) (Figure 7). SYTO 9 can diffuse through intact membranes, while PI can only enter bacterial cells through large pores or membrane holes. We observed the red fluorescence of PI in *L. lactis* cells treated with rombocin A, indicating membrane disruption similar to nisin. In contrast, nisin(1–22) treatment did not cause any significant membrane disruption, as only the green dye was observed. We further examined the pore-forming ability of rombocin A using potassium-ion-release experiments with potassium-ion-sensitive fluorescent probe PBFI (Figure 7B). Nisin caused an immediate increase in signal, indicating the release of intracellular potassium ions, whereas rombocin A and nisin(1–22) did not have this effect. Additionally, we evaluated the membrane potential of *L. lactis* cells treated with rombocin A, using the membrane potential-sensitive fluorescent probe DiSC3(5). The results showed that rombocin A caused significant membrane depolarization, similar to nisin (Figure 7C), while nisin(1–22) did not. Our findings suggest that rombocin A interacts with the membrane without forming stable pores and its membrane depolarization ability could contribute to bacterial cell killing.

### 2.6 Rombocin A Displays High Thermal and pH Stability and Resistance to Proteolytic Enzyme Degradation

**Figure 8:**
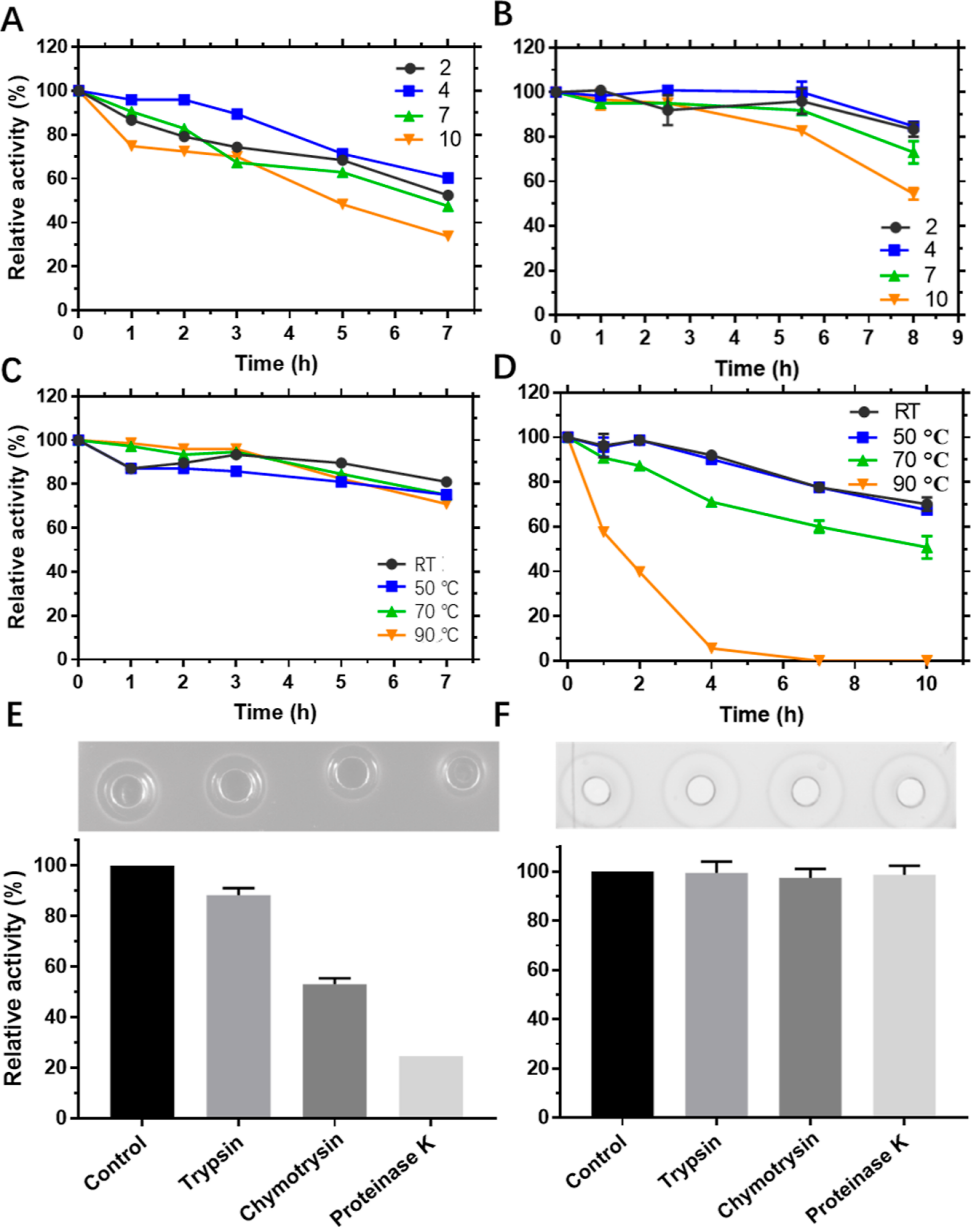
Stability of rombocin and nisin. (A) pH stability of nisin Z. (B) pH stability of rombocin A. (C) Thermal stability of nisin Z. (B) Thermal stability of rombocin A. (E) Relative antimicrobial activity of the nisin Z after exposure to different proteolytic enzymes. (F) Relative antimicrobial activity of the rombocin A after exposure to different proteolytic enzymes.

RiPPs have gained widespread applications, partly due to their stability under harsh conditions. To evaluate the stability of rombocin A, we subjected the peptide to different temperatures, pH values, and proteases (Figure 8). Rombocin remained stable for up to 5.5 h at different pH levels (2, 4, 7, and 10), with less than 20% reduction in activity. After 8 h of incubation, it still retained more than 80% of its activity at pH 2 and 4, and 70% at neutral pH (Figure 8B). Rombocin was stable for up to 10 h at temperatures between 22 and 50 °C, with less than 30% reduction in activity (Figure 8D). However, at high temperatures (90 °C), the antimicrobial activity decreased dramatically, with complete loss of activity after 7 h of incubation. Rombocin A also showed high resistance to proteolytic enzymes (Figure 8F). In contrast, nisin exhibited varying stability against trypsin, chymotrypsin, and proteinase K (Figure 8E). These results collectively demonstrate that rombocin has high thermal and pH stability and good resistance to proteolytic enzymes.

## 3 Discussion

Nisin, the most extensively studied bacteriocin produced by several *L. lactis* strains, has been utilized for food preservation in more than 80 countries. Its versatility is demonstrated by its effective use in diverse food products such as dairy items, canned goods, processed meats, fish, fruit juices, and beverages. With potent antimicrobial properties, nisin proves effective against a range of Gram-positive bacteria. Additionally, nisin has shown promising applications in the veterinary sector for the prevention and treatment of bovine mastitis.^5^ Up to 10 natural variants of nisin have been discovered in other bacterial species.^17^ In the current study, we have identified a novel short nisin variant, rombocin A, from the genome of *R. sedimentorium* RC002 that was isolated from chilled vacuum packed meat. From a practical point of view, the characterization of novel nisin variants may reveal desirable characteristics compared to nisin that increase or diversify the range of applications for the new bacteriocin in food processing. To expedite the characterization of bacteriocins, different approaches have been applied. Among these, heterologous expression systems have been developed offering several advantages over native systems, such as improved functional elucidation of the bacteriocin, better control over bacteriocin gene expression, and increased production levels.^22^ Here, we describe the heterologous expression and characterization of rombocin A, hence revealing its unique properties.

Multiple genes are required for nisin synthesis and immunity, and it has been shown that NisBTC can produce and export not only fully modified nisin but also a nonlantibiotic fusion of the leader peptide with dehydrated angiotensin.^23^ This broad substrate specificity of the nisin dehydrating and transport machinery suggests that lantibiotic enzymes could be utilized for the synthesis of a wide range of novel dehydroresidue-containing peptides or novel lantibiotic structures. Building upon these advantages, nisin has been bioengineered to enhance its antimicrobial activity, heat stability, solubility, diffusion, and protease sensitivity.^24,25^ In this study, we further expand the utility of nisin-controlled expression systems and NisBTC modification machinery, which uses a method for inducing modification enzymes in advance to enhance the peptide modification efficiency (Figure 3). The development of an efficient heterologous modification system offers significant advantages, facilitating the characterization and further modification of novel lantibiotics.

Maturation of rombocin A through in vitro cleavage of the nisin leader peptide demonstrated that it has a broad range of activity against Gram-positive strains (Figure 3E). Subsequent MIC determination revealed that while rombocin A was less active than nisin, it displayed selective antimicrobial activity against pathogenic *L. monocytogenes* (Table 1). It has been shown that engineering of nisin can result in variants with enhanced bioactivity or specific activity. Most notable are mutations N20P, M21V, and K22S, with enhanced bioactivity and specific activity against Gram-positive pathogens including *L. monocytogenes* and/or *S. aureus*.^24^ The specificity of rombocin A and its analogue rombocin K against *L. monocytogenes* (Table 1) suggests that they may be more suitable options than nisin for certain applications, especially since *L. monocytogenes* is among the most naturally nisin-resistant Gram-positive pathogens.^26^ Furthermore, the specificity of rombocin should be a good candidate as a drug agent.

Nisin exerts a dual mode of action against the target bacteria. First, it inhibits cell wall synthesis by displacing lipid II from the septa. Subsequently, it induces membrane pore formation through lipid II-induced transmembrane reorientation.^27^ The binding of lipid II occurs within the pyrophosphate cage, where rings A and B of nisin physically interact with the pyrophosphate moiety.^28^ The hinge region and rings D and E mediate the reorientation of nisin in membranes from parallel to perpendicular with respect to the membrane surface, thereby facilitating subsequent membrane pore formation.^29^ Structurally, rings A and B of rombocin A and nisin are conserved (Figure 2E). Consistent with its structure, the ability of rombocin A to also bind to lipid II has been demonstrated currently (Figure 5) and can be linked to the two rings. The time-killing assay is a widely used method to assess whether an antibiotic is bacteriostatic or bactericidal. In a time-killing assay, rombocin A was found to be bactericidal but slower than nisin (Figure 6). The bactericidal activity of rombocin A was further confirmed by fluorescence microscopy (Figure 7A). In spite of its bactericidal activity, the mode of action of rombocin A is not fully understood. One proposed mechanism of action is that it causes a pore forming ability similar to nisin. The pore forming ability was tested by potassium ion efflux assays (Figure 7B). In the leakage experiments, the nisin-induced potassium leakage was measured directly by using the dye, but this was not observed for rombocin A, which means the rombocin A’s ability of killing the cells uses a different mechanism. This is similar to the lantibiotic daptomycin where daptomycin kills bacteria by membrane permeabilization and depolarization.^30,31^ Here, we examined the effects of rombocin A on the membrane potential of the *L. lactis* strain (Figure 7C). Membrane depolarization was observed after rombocin A treatment, and this could account for the bactericidal effect of the lanthipeptide on target cells.

To ensure a diversified application, it is crucial for bacteriocins to exhibit stability under different conditions. Our investigation revealed that pH had a more significant impact on nisin than rombocin A (Figure 8A,B). Previous studies have shown that nisin is unstable at high pH, and degradation products of nisin involving dehydrated residues (such as Dha5 and Dha33) suggest that the integrity of unsaturated amino acids is a crucial factor affecting nisin’s chemical stability.^32^ The addition of a water molecule to the double bond of Dha can result in the formation of the corresponding amide and keto acid, leading to cleavage of the polypeptide chain at position 5 and/or in the C-terminal part at position 33. Notably, rombocin A lacks Dha at position 33 due to the absence of ring E, which enables it to avoid degradation at position 33. Additionally, rombocin A possesses one more Dhb located at ring C, which may contribute to its increased stability. Rombocin is less stable at high temperatures above 90 °C than nisin (Figure 8C,D), and the reason for this is not fully understood. It should be noted that the stability assay is based on residual biological activity. Some degradation products of nisin, e.g., nisin A(1–32), nisin A(1–22) retain slightly higher biological activity,^33^ while any degraded rombocin A may cause nonfunctional peptide due to the decreased peptide length. On the other hand, the short rombocin renders the peptide more resistant to proteolytic enzymes than nisin (Figure 8E,F), as most of the residues in rombocin A reside in the macrocycles. The remarkable stability of rombocin A at neutral pH and its resistance to proteolytic enzymes provide significant advantages over nisin, given these limitations have hindered nisin’s use as a biopreservative, for example, in dairy products.^34^

Lantibiotics are gene-encoded, a feature that makes them amenable to bioengineering. Mutagenesis of lantibiotics has been widely performed to improve their antimicrobial and physicochemical properties.^25^ Nisin Z is cationic due to the presence of four positively charged residues (K12, K22, K34, and H31) and the absence of negatively charged equivalents. The importance of positive charge is given to the initial attraction of many cationic peptides to the cell envelope. To date, the effect of manipulating the charge of nisin has resulted in variable outcomes. This may be a consequence of the location at which the charged residues are incorporated. For instance, the introduction of negatively charged residues into the hinge region has had a detrimental impact (N20D, N20E, M21E, K22D, and K22E), whereas the introduction of positively charged residues has had a more beneficial impact on activity (N20K and M21K).^35^ In this study, the substitution of K25 at the end of the peptide with Ala abolished 30% of the antimicrobial activity (Figure 4C), which is consistent with the importance of a terminal Lys in lantibiotics whereby it interacts with the target membrane components, in particular with negatively charged lipids. Mutating the I12, a residue that is predicted to serve as a smaller flexible region between rings B and C, to Lys in rombocin A shows a significant increase (13%) in antimicrobial activity against *L. monocytogenes* (Figure 4C). This result is in contrast with previous nisin studies, which showed that mutant K12 to Ile caused 11% activity lost,^36^ an indication that the residue at position 12 plays different functional roles in both peptides. In all known Class I lanthipeptides the residue at position 9 is Pro apart from cesin A,^37^ which like rombocin A, possesses an Ala at this position. The P/A mutant in rombocin A decreased the antimicrobial activity of the peptide (Figure 4C). The conservation of P9 in Class I lanthipeptides emphasis its role in lipid II binding making it one of the least hotspots for natural induced mutagenesis.^27^ The current study suggests that rombocin A requires a smaller amino acid at this position most likely for conformational reasons that enhance binding to lipid II. Collectively, these data demonstrate that rombocin A is a good candidate for further modification through bioengineering whereby novel analogues can be created to further study the functionality of the bacteriocin with a focus on future development as an antimicrobial agent.

In conclusion, rombocin A is the first discovered Class I lanthipeptide that possesses the conserved rings A, B, C, and D of natural nisin variants, while lacking the essential ring E in the C-terminal domain. The short lanthipeptide shows selective antimicrobial activity against pathogenic *L. monocytogenes*, an important foodborne pathogen and displays activity against multidrug-resistant strains. Although devoid of the fifth macrocycle in the C-terminal part, rombocin A shows two inhibition modes of action mechanisms like nisin by binding to lipid II and impairs membrane functions. Rombocin A however does not result in intracellular loss of potassium ions like nisin. Rombocin A also possesses a high stability at different temperatures and pH values and is more resistance to proteolytic enzyme degradation compared to nisin, making rombocin A more appealing for application in the biopreservation of foods that are frequently contaminated by *L. monocytogenes*. The current study also demonstrates the suitability of rombocin A for bioengineering after generating novel derivatives, most notably mutant I/K that has enhanced bioactivity. Further characterization of rombocin A including in vivo and in vivo toxicity assays and further specific engineering as well as determining the detailed response of target bacteria will improve the efficacy of this novel lantibiotic for diverse applications.

## 4 Materials and Methods

### 4.1 Chemicals and Reagents

Enzymes used for molecular biology experiments were obtained from Thermo Fisher Scientific (Waltham, MA). Unless specified, all reagents were procured from Sigma-Aldrich (St. Louis, MO). Nisin Z was obtained from Handary (Brussel, Belgium). Fluorescent dyes DiSC3(5) were obtained from Thermo Fisher Scientific (Waltham, MA). The LIVE/DEAD Bacterial Viability Kit was purchased from Invitrogen, and Abcam (Waltham, USA) supplied the fluorescent dye PBFI.

### 4.2 Bacterial Strains, Plasmids, and Growth Conditions

The bacterial strains and plasmids used in this study are listed in Table S2. *R. sedimentorum* strains were cultivated anaerobically at 8 °C for 10 days on Colombia agar supplemented with 5% sheep blood agar. *L. monocytogenes*, *B. cereus*, and *S. aureus* strains were grown overnight in Luria–Bertani (LB) medium at 37 °C with constant shaking at 220 rpm. *Enterococcus* strains were cultured at 37 °C in M17 broth (Oxoid) supplemented with 0.5% (w/v) glucose (GM17) while being shaken at 220 rpm for 12 h. All *L. lactis* strains were cultivated at 30 °C in GM17. The media were supplemented with 5 μg/mL chloramphenicol (Cm) or erythromycin (Em) as needed. *L. lactis* NZ9000 was used as the host for cloning, plasmid maintenance, and peptide expression. Protein expression was carried out in the minimal expression medium (MEM).

### 4.3 Molecular Biology Techniques

Table S3 provides a list of primers used for PCR and sequencing, all of which were purchased from Biolegio B.V. (Nijmegen, The Netherlands). The cloning work was performed according to a previously described protocol.^38^

### 4.4 In Silico-Based Identification of *R. sedimentorum* Strains

Two strains, RC001 and RC002, were isolated anaerobically from chilled vacuum-packed meat as described in a previous study aimed at isolating and identifying *Clostridium estertheticum* complex (CEC) strains.^39^ The strains could neither be identified as members CEC nor *Clostridium* sensu stricto group; hence, they were subjected to in silico-based identification methods as follows. Microbial genomic DNA was extracted from fresh cultures using the DNeasy Blood and Tissue Kit (Qiagen, Hilden, Germany). Sequencing libraries, whose length was 150–300 bp, were prepared using the Illumina Nextera DNA Flex and sequenced on an Illumina MiniSeq (Illumina, San Diego, CA, United States) with a minimal coverage of 50×. The MiniSeq MidOutput Reagent Cartridge (300 cycles) was used. Demultiplexing and adapter trimming were done using the Miniseq local run manager version 2.4.1 using standard settings. The reads were checked for quality using FastQC and then assembled with SPAdes v. 3.12.0 using Shovill 1.0.9.2. The quality of the assembled genomes was checked by using CheckM. 16S rRNA sequences (1216 bp) of the strains were extracted from the respective genomes in silico using ContEST16S. The strains were identified in silico using a 16S-Based ID tool, which allows bacterial strains to be identified by their 16S rRNA sequences using the EZBioCloud Database. Curated 16S rRNA sequences (1216–1217 bp) of six Type strains of validly published *Romboutsia* species namely *R. sedimentorum* LAM201^T^, *R. ilealis* CRIB^T^, *R. weinsteinii* CCRI-19649^T^, *R. maritimum* CCRI-22766^T^, *R. lituseburensis* ATCC 25759^T^, and *R. hominis* FRIFI^T^ were downloaded from the EZBioCloud Database. All sequences were aligned in CLC Workbench Genomics v. 8.1 (Qiagen, Aarhus, Denmark) using the progressive alignment algorithm with default settings and a phylogenetic tree created from the aligned sequences in the CLC Workbench Genomics using the Maximum likelihood Phylogeny method. The tree was constructed using the UPGMA method by applying the Jukes Cantor model. Bootstraps were based on 1000 replicates. The 16S rRNA sequence of *C. estertheticum* DSM 8809^T^ (1220 bp) was used as the outgroup.

### 4.5 Genome Mining for Bacteriocin Biosynthetic Gene Clusters

The genomes of strains RC001 and RC002 were mined for bacteriocin gene clusters using antiSMASH v.6^40^ and BAGEL4.^41^ The amino acid sequence of an identified nisin-like precursor peptide was downloaded from a gene cluster identified by both tools. The amino acid sequence of nine known natural nisin variants were downloaded from the BACTIBASE Web server in February 2023 or identified through literature search. All sequences were aligned, and a phylogenetic tree was created from the aligned sequences in the CLC Workbench Genomics as described above.

### 4.6 Precursor Peptide Expression and Precipitation

For the strain *L. lactis* NZ9000 carrying plasmids pIL3eBTC and pNZ-rombocin, a single colony was used to inoculate 4 mL of GM17CmEm broth, which was then incubated overnight at 30 °C. The culture was subsequently used to inoculate 20 mL of MEM at a 40-fold dilution, and when the OD_600_ reached 0.4–0.6, 10 ng/mL nisin was added to induce the expression of both the peptide and modification machinery NisBTC. After 3 h of induction at 30 °C, the supernatant of cultures was collected by centrifugation at 8000*g* for 20 min. Similarly, for the strain *L. lactis* NZ9000 carrying plasmids pTLReBTC and pNZ-rombocin, the overnight culture was used to inoculate 20 mL of GM17CmEm broth (40-fold), and when the OD_600_ reached 0.4–0.6, and 0.5 mM Zn^2+^ was added to induce the NisBTC. After 3 h of induction, the medium was replaced with an equal volume of fresh MEM media. Subsequently, 10 ng/mL nisin was added to induce the expression of the peptide, followed by another 3 h of incubation at 30 °C. The supernatant of cultures was then collected as described above. To further analyze the precursor peptides, they were precipitated using trichloroacetic acid (TCA), as described by Link and LaBaer.^42^ The resulting dried pellets were either stored at 4 °C or suspended in 0.4 mL of a 0.05% aqueous acetic acid solution for further analysis.

### 4.7 Preliminary Characterization and Purification of Rombocin

#### 4.7.1 Tricine-SDS-PAGE Analysis

The precipitated precursor peptides were subjected to Tricine-SDS-PAGE analysis, following the previously described protocol.^38^

#### 4.7.2 MALDI-TOF Mass Spectrometry Characterization

Matrix-assisted laser desorption ionization-time-of-flight (MALDI-TOF) mass spectrometry analysis was carried out using a 4800 Plus MALDI TOF/TOF analyzer (Applied Biosystems) in the linear positive mode at the University of Groningen as a previously described protocol.^38^ The mass accuracy obtained in linear mode measurements was estimated as ±1‰.

#### 4.7.3 Peptide Purification

To obtain pure peptides for activity tests and mode of action studies, the supernatant from 1 L culture was first incubated with purified NisP^43^ at 37 °C for 6 h to remove the nisin leader. The resulting supernatant was then loaded onto a C18 open column (Spherical C18, particle size: 40–75 μm, Sigma-Aldrich), washed, and eluted with different concentrations of buffer B (buffer A, Milli-Q with 0.1% TFA; buffer B, acetonitrile with 0.1% TFA). Active fractions were identified and lyophilized and then further purified by HPLC using an Agilent 1200 series HPLC with a RP-C12 column (Jupiter 4 μm Proteo 90A, 250 × 4.6 mm, Phenomenex). The fully modified peptide with the correct molecular weight was identified, lyophilized, and stored as powder until further use.

### 4.8 Characterization of Physiochemical Properties and Antimicrobial Activity of Rombocin

#### 4.8.1 Effects of Enzyme, pH, and Temperature on Antibacterial Activity

To investigate the impact of proteolytic enzymes, pH, and temperature on the antimicrobial activity, a representative strain of *L. lactis* 1363 was utilized. For the proteolytic enzyme assay, 40 μL of peptide (1 mg/mL) was incubated with or without final concentrations of 1 mg/mL proteolytic enzymes at room temperature for 3 h, followed by an activity test in the agar well. To determine pH stability, the pH was adjusted to 2, 4, 7, and 10 using 1 M HCl or NaOH. The temperature stability was assessed by incubating the peptide at 22, 50, 70, and 90 °C for a specified duration. All experiments were performed in triplicate.

#### 4.8.2 Agar Well Diffusion Assay and Mutant-Activity Test

Overnight cultures of the test strains were inoculated at a final concentration of 0.1% (v/v) into melted LB agar at 45 °C and then poured onto plates. After solidification, 8 mm wells were created and filled with 40 μL of the lantibiotic solution (1 mg/mL). The lantibiotics were activated by adding 5 μL of NisP directly to the well. The plates were then incubated overnight at 30 °C, and the zone of inhibition was measured. Zone diameters were calculated by subtracting the area of the well (π*r*^2^) from the area of the zone (π*r*^2^) and reported in millimeters. To determine the peptide concentration, purified and lyophilized peptides were resuspended in 0.05% aqueous acetic acid solution and quantified by HPLC following the protocol described by Schmitt et al.^44^ All experiments were performed in triplicate.

#### 4.8.3 Determination of the MIC

MIC values were determined using broth microdilution following standard guidelines^45^ and the MIC was determined as the lowest concentration of antibiotic that showed no visible growth. Each experiment was performed in triplicate.

### 4.9 Determination of Mode of Action of Rombocin

#### 4.9.1 Spot-on-Lawn Assay to Measure Peptide-Lipid II/LTA Complex Formation

To evaluate the binding of peptide and lipid II or lipoteichoic acid (LTA), an overnight cultured *L. lactis* MG1363 was added to 0.8% GM17 (w/v, temperature 45 °C) at a final concentration of 0.1% (v/v), and then, the mixture was poured onto 10 mL plates. The binding of peptide and lipid II or LTA was further evaluated by spotting of purified lipid II (300 μM, 2 μL) or LTA (1 mg/mL, 2 μL) to the edge of the inhibition halo of antibiotics. Briefly, antimicrobials were loaded to the agar plate. After the antimicrobial solution drops had dried, lipid II or LTA was spotted to the edge of inhibition halo of antimicrobials. The plates were then incubated overnight at 30 °C after the drops had dried.

#### 4.9.2 Time-Kill Assay

The bactericidal activity of nisin, nisin(1–22), and rombocin was evaluated according to a previously described procedure based on Guo et al.^38^ Briefly, an overnight culture of *L. lactis* MG1363 was diluted 50-fold in GM17 medium and incubated at 30 °C. The bacteria were grown until reaching an optical density at 600 nm (OD_600_) of 0.5, at which point the cell concentration was adjusted to 5 × 10^5^ CFU/mL. Subsequently, the bacteria were challenged with a 5-fold MIC of each peptide. A control sample of an untreated cell suspension was included. At specific time points, 50 μL aliquots were collected, and undiluted and 10-fold serially diluted suspensions were plated on GM17 agar. The plates were incubated overnight at 30 °C, and the resulting colonies were counted and calculated as colony-forming units per milliliter (CFU/mL). Both assays were performed in triplicate to ensure reproducibility.

#### 4.9.3 Fluorescence Microscopy Assay

*L. lactis* was cultured until it reached an OD_600_ of 0.8 and then pelleted at 8000*g* for 5 min and washed three times in 0.7% NaCl. The cell density was normalized to an OD_600_ of 0.4 in 0.7% NaCl, and a concentration of 5-fold MIC value of each tested antibiotic was added to the cell suspension simultaneously with SYTO 9 and propidium iodide using the LIVE/DEAD Baclight Bacterial Viability Kit (Invitrogen). After incubation at room temperature for 20 min, the compounds were removed by washing the cells with 0.7% NaCl. Finally, the cell suspensions were loaded onto 1.5% agarose pads and analyzed with a DeltaVision Elite microscope (Applied Precision).

#### 4.9.4 Potassium Ion Efflux Assays

To perform the K^+^ release assay, we used the K^+^-specific fluorescent probe PBFI. *L. lactis* was cultured in GM17 until the OD_600_ reached 0.6, after which the cells were harvested (5000*g*, 5 min) and washed twice with 10 mM HEPES (pH 7.2) with 0.5% glucose. The cells were then resuspended in the same buffer containing 10 μM PBFI. Data were analyzed using a Varioskan LUX Multimode Microplate Reader; cells were excited at 346 nm, and the fluorescence emission was measured at 505 nm to establish a baseline signal before the addition of 5-fold MIC antibiotics, after which data were collected. Nisin was used as a positive control.

#### 4.9.5 Determination of Membrane Potential

To measure the membrane potential, the membrane potential-sensitive fluorescent dye DiSC3(5) was utilized. *L. lactis* was cultured to an OD_600_ of 0.8, pelleted at 5000*g* for 5 min, and washed twice in 10 mM HEPES with 10 mM glucose (pH 7.2). The cell density was adjusted to an OD_600_ of 0.2 and loaded with 2 μM DiSC3(5) dye, followed by 20 min of incubation in the dark to stabilize the probe fluorescence. Next, the cell suspension was added to a 96-well microplate and incubated for 5 min with 100 mM KCl. Afterward, antibiotics were added at a final concentration of 5× MIC, and the fluorescence was monitored for 25 min. The fluorescence spectrometer’s excitation and emission wavelengths were adjusted to 622 and 670 nm, respectively. Three technical replicates were performed, and the representative example is shown.

## Data Availability

The genomes have been deposited in the NCBI under the bioproject PRJNA976091 (https://www.ncbi.nlm.nih.gov/bioproject/PRJNA976091). All data supporting the findings of this study are available within the paper and its Supporting Information files.

## Supporting Information Available

MALDI-TOF MS analysis of C18 open column purified peptides; ring analysis with the NEM MALDI-TOF spectrum of the peptide; MALDI-TOF MS analysis of HPLC purified peptides; spot-on-lawn assay used to assess the bacteriostatic or bactericidal activity of the peptide; theoretical average masses of different peptides produced in this study; plasmids and strains used in this study; primers used in this study; and supplementary methods (PDF)
