# Supplementary material for: Rombocin, a Short Stable Natural Nisin Variant, Displays Selective Antimicrobial Activity against Listeria monocytogenes and Employs a Dual Mode of Action to Kill Target Bacterial Strains
Source: ACS Synth Biol. 2024 Jan 9;13(1):370–83. doi: 10.1021/acssynbio.3c00612 (PMC10804407; doi:10.1021/acssynbio.3c00612)
Supplement: Supplementary file 1 — sb3c00612_si_001.pdf [file sb3c00612_si_001.pdf]

Supporting Information for

Rombocin, a short stable natural nisin variant, displays selective antimicrobial activity against *Listeria monocytogenes* and employs a dual mode of action to kill target bacterial strains

Longcheng Guo[a], Joseph Wambui[b], Chenhui Wang[a], Jaap Broos[a], Roger Stephan[b] and Oscar P. Kuipers[a, *]

a Department of Molecular Genetics, Groningen Biomolecular Sciences and Biotechnology Institute, University of Groningen, Groningen, The Netherlands

b Institute for Food Safety and Hygiene, Vetsuisse Faculty, University of Zurich, Zurich, Switzerland

***** Correspondence to Oscar P. Kuipers, o.p.kuipers@rug.nl

Running title: Rombocin, a short stable variant of nisin

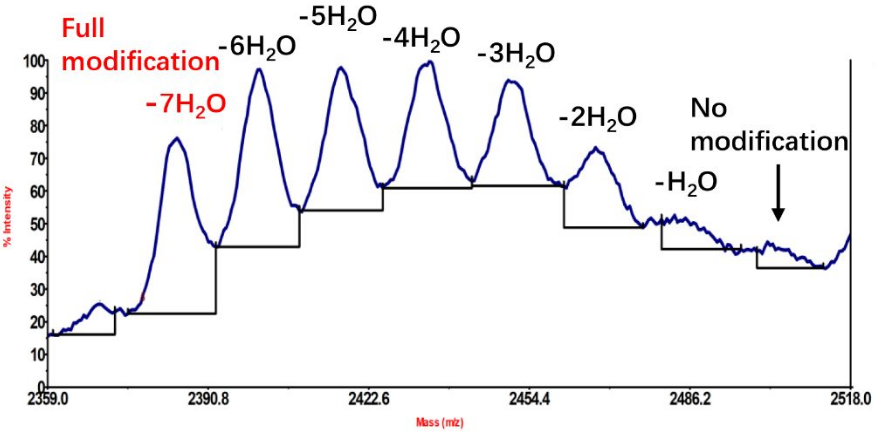

Figure S1 MALDI-TOF MS analysis on peptides purified from a 1L medium using the expression system outlined in Figure 3B1 and processed through C18 open column purification. The presence of peptides dehydrated at various times complicates subsequent purification efforts.

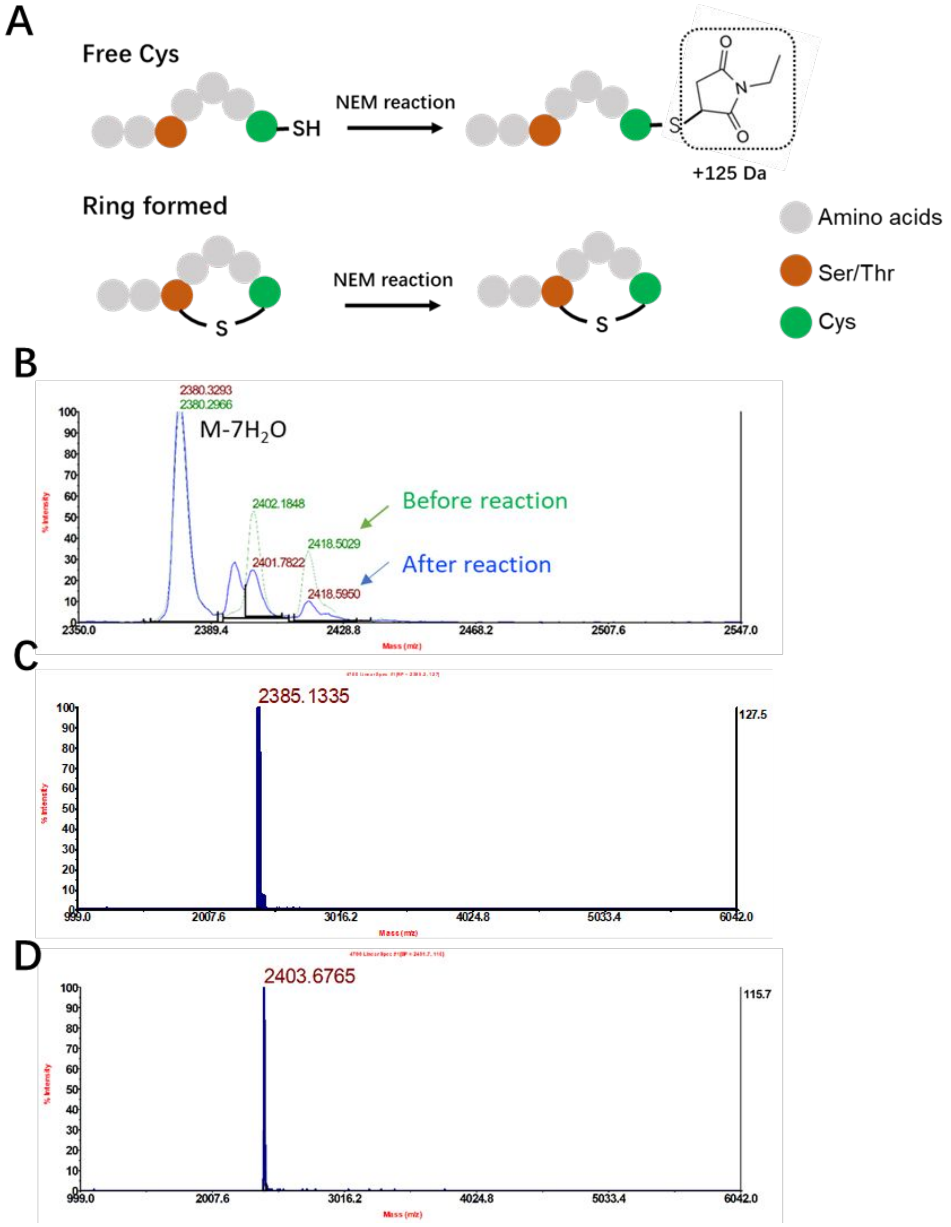

Figure S2 (**A**) A schematic illustration depicting the reaction of N-ethylmaleimide (NEM) with free Cys. If there is a Cys in the protein that does not form a thioether crosslink, a complete -SH group will be present on the peptide. Upon alkylation with NEM, the resulting product will have a larger molecular mass (125 Da) than the substrate. (**B**) NEM alkylation assay to determine the level of cyclization. The mass of the major peak after addition of NEM was consistent with the mass of the peptide before addition of NEM confirming the peptide lacked free Cys. (**C**) MALDI-TOF MS analysis of HPLC purified fully modified rombocin A. (**D**) MALDI-TOF MS analysis of HPLC purified fully modified rombocin K.

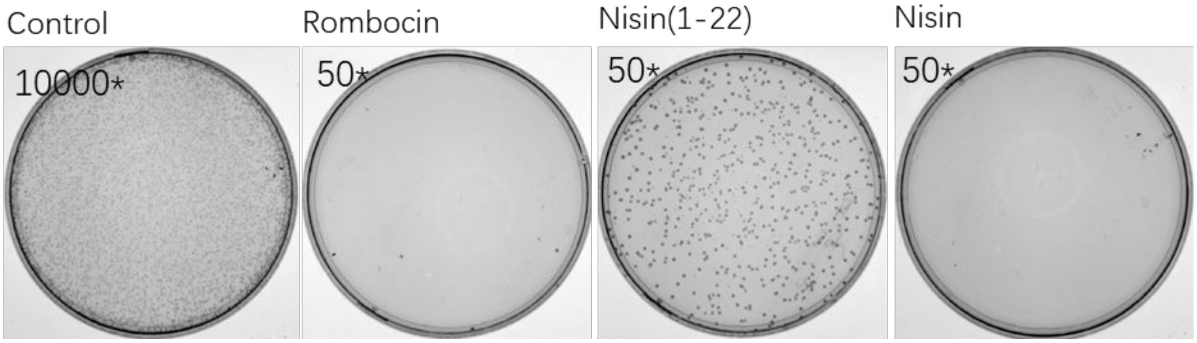

Figure S3 A spot-on-lawn assay used to assess the bacteriostatic or bactericidal activity of rombocin after 18 hours of incubation. *L. lactis* was subjected to a 5-fold MIC of the lantibiotics, with nisin serving as a bactericidal control and nisin(1-22) as a bacteriostatic control. The numbers in the image represent the dilution times of solutions used to spray the plates.

Table S1 Theoretical average masses of different peptides produced in this study.

peptide	modification	theoretical mass (Da)	found mass (Da)	difference	figure
Rombocin A	with leader sequence, 7 times dehydration	4715,64	4716	0,36	Figure 3D1
Rombocin A	with leader sequence, 6 times dehydration	4733,64	4735,39	1,75	Figure 3D1
Rombocin A	with leader sequence, 7 times dehydration	4715,64	4719,94	4,3	Figure 3D2
Rombocin A	7 times dehydration	2383,05	2385,13	2,08	Figure S2C
Rombocin K	7 times dehydration	2401,06	2403,67	2,61	Figure S2D

Table S2 Strains and plasmids used in this study.

Strains or plasmids	Characteristics	Reference
**Strains**		
Romboutsia sedimentorum RC001	Genome mining for bacteriocin gene clusters	This study
Romboutsia sedimentorum RC002	Genome mining for bacteriocin gene clusters	This study
Lactococcus lactis NZ9000	Plasmid construction, plasmid maintenance, and peptide expression	Kuipers et al., 1997
Lactococcus lactis MG1363	Indicator strain	Lab collection
*Listeria monocytogene*s LMG10470	Indicator strain	Lab collection
Listeria monocytogenes TT82E	Indicator strain	Lab collection
Listeria monocytogenes LK132	Indicator strain	Lab collection
Bacillus cereus CH-85	Indicator strain	Lab collection
Staphylococcus aureus LMG10147	Indicator strain	Lab collection
Staphylococcus aureus LMG15975	Indicator strain, methicillin resistant (MRSA)	Lab collection
Enterococcus faecium LMG11423	Indicator strain	Lab collection
Enterococcus faecium LMG16003	Indicator strain, vancomycin resistant (VRE)	Lab collection
Enterococcus faecalis LMG16216	Indicator strain, vancomycin resistant (VRE)	Lab collection
**Plasmids**		
pil3eBTC	*nisBTC*, encoding nisin modification machinery, under P_{nisA}, Ery^R	van Heel et al., 2013
pTLReBTC	*nisBTC*, encoding nisin modification machinery, under P_{czcD}, Ery^R	Guo et al., 2023
pNZ-nisA	*nisA*, encoding nisin A, under the control of P_{nisA}, Cm^R	van Heel et al., 2013
pNZ-rombocinA	*rombocinA*, encoding rombocin A, under the control of P_{nisA}, Cm^R	This study
pNZ-rombocinA(A/P)	Rombocin mutation, core peptide Ala9 changed to Pro	This study
pNZ-rombocinA(I/K)	Rombocin mutation, core peptide Ile12 changed to Lys	This study
pNZ-rombocinA(K/A)	Rombocin mutation, core peptide Lys25 changed to Ala	This study
pNZ-rombocinA(M/NMK)	Rombocin mutation, core peptide Met20 changed to Asn-Met-Lys	This study
pNZ-rom/nisin-tail	Rombocin and nisin hybrid peptide, core sequence ITSISLCTAGCITGVIMTCNMKTATCHCSIHVSK	This study

Table S3 Primers used in this study.

Name	Template[a]	Primer	Nucleic acid sequence (5' to 3')
pNZ-rombocinA	pNZ-nisA	pNZ-1	TGTATGAGTAACGGTTGTAAATAAGCTTTCTTTGAACCAAAATTAG
		pNZ-2[b]	TGTCATAATTACTCCTGTAATACAACCTGCTGTACATAGCGAAATACTTGTAATGC
pNZ--romA(A/P)	pNZ-romA	pNZ-3	CCAGGTTGTATTACAGGAGTAATTATGACA
		pNZ-4[b]	TGTACATAGCGAAATACTTGTAATGCGT
pNZ-romA(I/K)	pNZ-romA	pNZ-5	GCAGGTTGTAAAACAGGAGTAATTATGACATGTA
		pNZ-4[b]	TGTACATAGCGAAATACTTGTAATGCGT
pNZ-romA(K/A)	pNZ-romA	pNZ-6[b]	ACGGTTGTGCATAAGCTTTCTTTGAACC
		pNZ-7	TACTCATACATGTCATAATTACTCCTGTAATACAACC
pNZ-romA(M/NMK)	pNZ-romA	pNZ-8	ATGAAAAGTAACGGTTGTAAATAAGCTTTC
		pNZ-9[b]	GTTACATGTCATAATTACTCCTGTAATACAA
pNZ-rom/nisin-tail	pNZ-romA	pNZ-10	CATTGTAGTATTCACGTAAGCAAATAAGCTTTCTTTGAACCAAAATTAG
		pNZ-11[b]	ACAAGTTGCTGTTTTCATGTTACATGTCATAATTACTCCTGTAATAC
pNZ-sequencing		pNZ-12	TATGAGATAATGCCGACTGTACTTTTTAC

[a]The romA here refer to as rombocin A . [b] The primer 5’ end was phosphorylated.

Supplementary methods

Screening the antibacterial activity of peptides by spot-on-lawn assay

To prepare the plates, an overnight culture of the strains was added to 0.8% LB agar or GM17agar (wt/vol) at 45 ℃ and mixed to a final concentration of 0.1% (vol/vol) before being poured onto the plates (10 ml each). Next, 5 µl of the precursor peptide and 1 µl of 1 mg/mL NisP were dropped onto the plates and left to dry before incubation. The plates were then incubated overnight at 37°C, except for *L. lactis*, which was incubated at 30°C.

Evaluation of (methyl)lanthionine formation

16 µL of phosphate-buffered saline (pH 7.4) was added to the samples followed by treatment with 2 µL of 5 mg/mL tris[2-carboxyethyl]phosphine for 30 min at room temperature. Next, 4.5 µL of 25 mg/mL N-ethylmaleimide was added to the samples, which were then incubated at room temperature for 2 h. Subsequently, the samples were desalted using a C18 ZipTip (Millipore) according to the manufacturer's instructions and subjected to MALDI-TOF MS analysis.

Growth curve and lipid II/LTA binding assay

Overnight cultures of *L. lactis* MG1363 were diluted to an OD_{600} of 0.05 and added to a 96-well plate, which was incubated at 30°C. When the OD_{600} reached 0.1, 5 × MIC peptide was added to each well. To investigate the association with peptides, 2 µl of lipid II or LTA was added. The growth curve was recorded over the following 6 hours using a microplate spectrophotometer under the same conditions, with three replicates used for each treatment.

References

Guo, L., Wang, C. H., Broos, J., Kuipers, O. P. (2023). Lipidated variants of the antimicrobial peptide nisin produced via incorporation of methionine analogs for click chemistry show improved bioactivity. *Journal of biological chemistry.* 299(7), 104845..

Kuipers, O. P., de Ruyter, P. G., Kleerebezem, M., de Vos, W. M. (1997). Controlled overproduction of proteins by lactic acid bacteria. *Trends in biotechnology*, *15*(4), 135-140.

van Heel, A. J., Mu, D., Montalbán-López, M., Hendriks, D., Kuipers, O. P. (2013). Designing and producing modified, new-to-nature peptides with antimicrobial activity by use of a combination of various lantibiotic modification enzymes. *ACS synthetic biology*, *2*(7), 397-404.
